# Multi-Omics Reveals Molecular and Genetic Mechanisms Underlying Egg Albumen Quality Decline in Aging Laying Hens

**DOI:** 10.3390/ijms26167876

**Published:** 2025-08-15

**Authors:** Mingyue Gao, Junnan Zhang, Ning Yang, Congjiao Sun

**Affiliations:** 1 Frontier Science Center of Molecular Design Breeding, State Key Laboratory of Animal Biotech Breeding, China Agricultural University, Beijing 100193, China; gaomingyue010530@163.com (M.G.); cauzhangjn@163.com (J.Z.); nyang@cau.edu.cn (N.Y.); 2 National Engineering Laboratory for Animal Breeding, College of Animal Science and Technology, China Agricultural University, Beijing 100193, China

**Keywords:** scRNA-seq, Haugh unit, laying hens, genomics, transcriptomics

## Abstract

As the laying cycle is prolonged, the egg albumen quality exhibits a declining trend. A Haugh unit (HU) is a standard measure of the albumen quality, which reflects viscosity and freshness. During the late laying period, the HU not only decreased significantly, but also exhibited greater variability among individuals. The magnum, as the primary site of albumen synthesis, plays a central role in this process; however, the mechanisms by which it regulates the albumen quality remain unclear. To address this, we obtained genomic and transcriptomic data from 254 individuals, along with single-cell RNA sequencing (scRNA-seq) data of the magnum tissue. Genome-wide association studies (GWAS) across five laying stages (66, 72, 80, 90, and 100 weeks of age) identified 77 HU-associated single-nucleotide polymorphisms (SNPs). Expression quantitative trait locus (eQTL) mapping linked these variants to the expression of 12 genes in magnum tissue. In addition, transcriptomic analysis using linear regression and random forest models identified 259 genes that significantly correlated with the HU. Single-cell RNA sequencing further revealed two key cell types, plasma cells and a subset of epithelial cells, marked by *ADAMTSL1* and *OVAL*, which are functionally relevant to the HU. Through integrated Transcriptome-Wide Association Study (TWAS) and Summary-data-based Mendelian Randomization (SMR) analyses, we identified four robust regulators of the albumen quality: *CISD1*, *NQO2*, *SLC22A23*, and *CMTM6*. These genes are functionally involved in mitochondrial function, antioxidant defense, and membrane transport. Overall, our findings uncovered the genetic and cellular mechanisms underlying age-related decline in the albumen quality and identified potential targets for improving the egg quality in aging flocks.

## 1. Introduction

The reproductive system of laying hens plays a crucial role in determining both the internal and external egg quality. Unlike mammals, the avian reproductive tract does not support embryonic development, but rather facilitates the stepwise formation of egg components [1]. Eggs are widely recognized as functional foods owing to their rich protein composition [2]. Among these, albumen serves as a primary source of essential nutrients and functions as a critical antimicrobial barrier to protect developing embryos from pathogenic infections [3]. Among the oviduct segments, the magnum serves as the primary site for albumen secretion, a process requiring approximately three hours, making it a critical determinant of the egg quality [4,5]. The Haugh unit (HU) is a key indicator of the albumen quality and is calculated based on the egg weight and the height of the thick albumen. As a standardized and widely accepted metric, HU reflects the viscosity and freshness of the albumen, with higher values indicating thicker albumen and superior freshness. Notably, the HU also correlates strongly with hatchability rates, making it a comprehensive indicator of both the egg quality and reproductive potential [6].

Driven by industrial demands, modern egg production has progressively extended laying cycles, with current targets aiming for 500 high-quality eggs per hen by 100 weeks of age [7]. However, with the laying cycle lengthening, both the productive performance and tissue function decline, eventually compromising the egg quality [8,9,10,11]. Research has demonstrated that aging laying hens exhibit a decline in the antioxidant capacity, rendering them more susceptible to oxidative stress [12]. Notably, with advancing age, the thick albumen proportion, thick-to-thin albumen ratio, albumen height, and HU showed an initial increase followed by a significant decline [13].

The quality of albumen proteins is influenced by various factors, including the genetic background, age, and environmental conditions [14]. Studies have revealed that aged laying hens exhibit smaller yet more structurally complex mucosal folds in the magnum, along with significantly shorter secondary folds [15]. Concurrently, both the mRNA and protein expression profiles of albumen constituents critically influence the egg quality [16]. During the late laying phase, laying hens exhibit decreased RNA splicing efficiency, which subsequently leads to a significant downregulation of ovomucin expression [17].

However, the specific molecular mechanisms responsible for age-related decline in the albumen quality remain unclear. To address this, we adopted a comprehensive multi-omics strategy that integrates large-scale genomic and transcriptomic data. By performing expression quantitative trait locus (eQTL) mapping, transcriptome-wide association study (TWAS), and summary-data-based Mendelian randomization (SMR) analysis in magnum, we systematically identified regulatory variants that link genetic variation to gene expression and ultimately to the HU. Following this, we performed single-cell RNA sequencing to resolve the cellular context of gene regulation, enabling the assignment of candidate genes to specific cell populations within the magnum. This integrative approach not only enhances the resolution of regulatory mechanism analysis, but also provides novel insights into the cell-type-specific basis of albumen quality deterioration. Our findings may inform the development of targeted strategies to improve and maintain the egg quality during extended laying cycles.

## 2. Results

### 2.1. Integrated GWAS and eQTL Analysis Identifies Functional Loci for HU

To explore the genetic regulation of the HU during the extended egg-laying cycle, we performed genome-wide association studies (GWAS) based on HU measurements collected at 66, 72, 80, 90, and 100 weeks of age from 254 Rhode Island Red hens. Overall, 77 single-nucleotide polymorphisms (SNPs) were significantly associated with the HU (Table S1). The results showed that significant SNPs formed distinct peaks at different time points with no overlapping regions across ages, suggesting that the HU is a polygenic trait controlled by multiple loci scattered throughout the genome.

The results showed that distinct genomic regions were associated with HU at different time points. At 66 weeks of age, seven significant SNPs were found within a 3.8 Mb region (20.2–20.5 Mb) on chromosome 3. At 72 weeks of age, 30 significant SNPs clustered in a 2 Mb region (65.7–67.6 Mb) on chromosome 2. At 80 weeks of age, 10 significant SNPs were located in a narrow region (90.4–90.5 Mb) on chromosome 4. In contrast, the number of significant SNPs decreased at 90 and 100 weeks of age, possibly due to increased phenotypic heterogeneity in the later laying stages. Therefore, we integrated GWAS results from all five time points to identify candidate regions involved in HU regulation during the late laying period.

**Figure 1:**
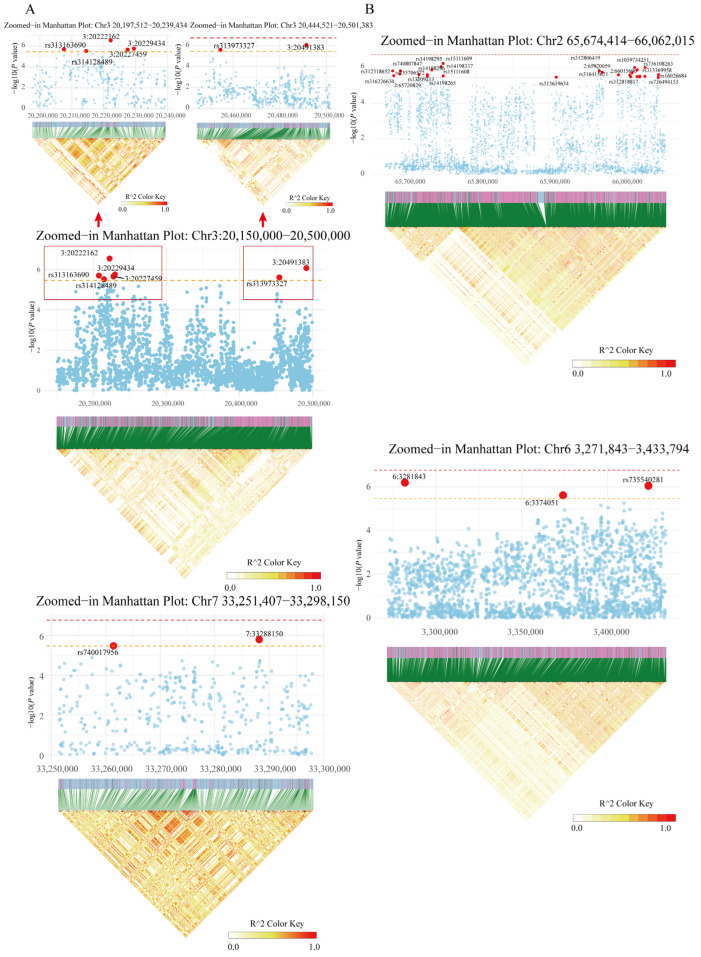
Regional plots of significant association signals from GWAS. (**A**,**B**) show magnified views of prominent genomic regions corresponding to the red boxed areas in the Manhattan plots for Haugh unit (HU) at 66 and 72 weeks of age, respectively. Each panel includes a Manhattan plot (upper) and the corresponding linkage disequilibrium (LD) heatmap (lower). In the Manhattan plots, two dashed horizontal lines indicate the genome-wide significance threshold (*p* < 1.74 × 10^−7^) and the suggestive significance threshold (*p* < 3.48 × 10^−6^), with red dots indicating significant loci. In the LD heatmaps, color intensity reflects the strength of pairwise linkage disequilibrium, with red indicating stronger LD.

We then selected representative peaks for further regional analysis using linkage disequilibrium (LD) mapping (Figure 1 and Figure S1). The resulting LD heatmaps revealed strong correlations among SNPs within the same haplotype blocks. Based on the LD structure visualized using LDBlockShow (v 1.39), we expanded the 77 significant SNPs by including those located within the same LD blocks, as defined by the software’s default settings, thereby generating a broader candidate SNP set for downstream analysis (Table S2). Functional validation through eQTL analysis showed that two genes, ENSGALG00010011348 (*SLC22A23*) and ENSGALG00010011426 (*NQO2*), were significantly regulated by cis-eQTLs. Additionally, trans-eQTL analysis identified 10 genes under distal regulation by these significant variants (Table S3).

### 2.2. Identification of Key Genes Related to HU in the Magnum Employing Linear Regression and Random Forest Analysis

**Figure 2:**
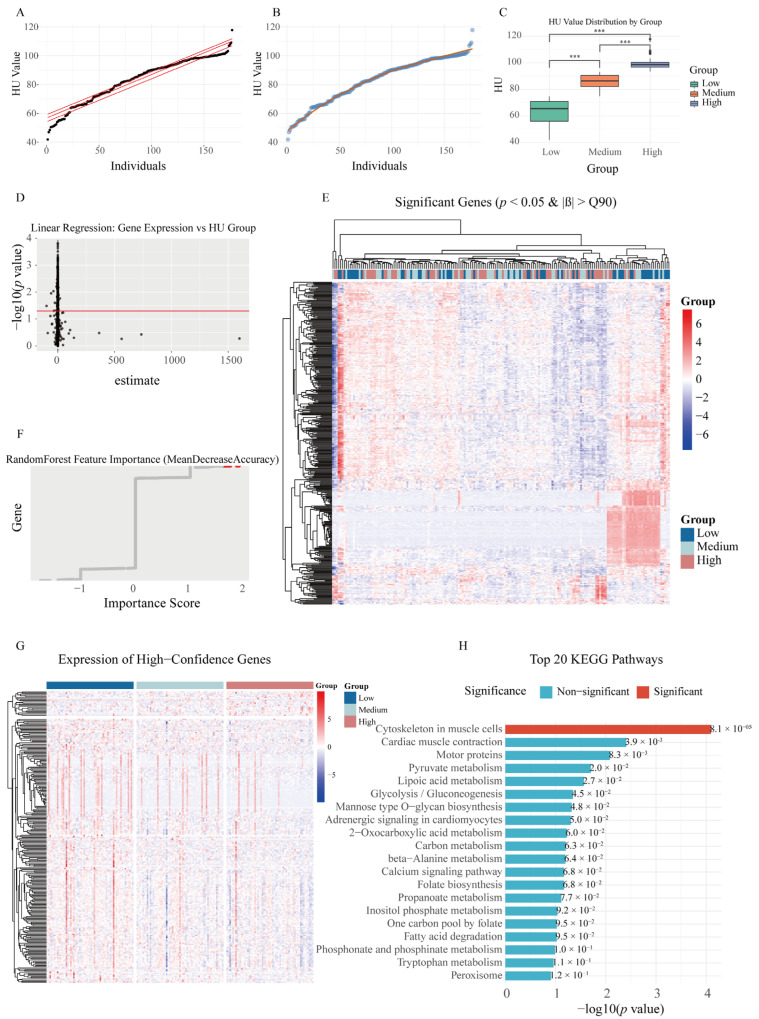
Identification of Haugh unit (HU)-associated genes via phenotypic stratification and transcriptomic analyses. (**A**,**B**) Distribution of HU values across 177 samples. Red lines indicate the 25th and 75th percentiles (**A**) and the fitted trend line (**B**) of the phenotype. (**C**) Tukey’s HSD test results for HU among phenotype-based groups. *** *p* < 0.001. (**D**) Distribution of gene effect estimates from linear regression analysis; genes above the red threshold line are considered significant. (**E**) Expression heatmap of 399 genes in 177 samples. Dark blue indicates the low-HU group, light blue the medium-HU group, and red the high-HU group. (**F**) Result of random forest analysis. The importance score reflects the contribution of each gene to the HU phenotype; genes in red represent the top 10% of importance. (**G**) Expression heatmap of 206 genes in 177 samples. (**H**) Results of KEGG pathway enrichment analysis. Red bars indicate significantly enriched pathways, while blue bars denote non-significant pathways.

To investigate the gene expression differences associated with varying HU phenotypes at 100 weeks of age and further evaluate the impact of key gene expression changes on the egg-laying performance, we conducted transcriptome analysis. The results showed that the HUs were primarily distributed between the 0.25 and 0.75 quantiles, exhibiting a relatively uniform distribution (Figure 2A,B). Based on the HU, the cohort was stratified into three groups (low/mid/high). Tukey’s HSD test confirmed statistically significant intergroup differences (*p* < 0.001; Figure 2C).

First, we performed linear regression analysis on the grouped individual data. The results showed that the estimated values for most genes were distributed on both sides of zero, indicating relatively stable expression trends for the majority of genes during the increase in the HU (Figure 2D). Based on this, we identified 399 genes meeting the criteria of *p*-value < 0.05 and estimates > 90%, suggesting these genes exhibited expression patterns strongly associated with the HU. Subsequently, we generated an expression heatmap of these 399 genes, which revealed distinct expression differences among the various HU groups (Figure 2E).

To further refine the selection of HU-associated genes, we employed a random forest model to analyze the same dataset and selected the top 10% most important genes (2641 genes) (Figure 2F). By intersecting these with the 399 genes obtained from linear regression analysis, we ultimately identified 259 candidate genes showing significant association with the HU (Table S4).

Finally, we further analyzed the 259 candidate genes by focusing on the 206 genes that possessed annotated gene symbols, which were then visualized in heatmaps and analyzed through KEGG enrichment (Figure 2G,H). The results revealed enrichment in several biological pathways, including cytoskeletal organization in muscle cells, motor protein activity, pyruvate metabolism, folate biosynthesis, and calcium ion signaling pathways, suggesting that these processes may be functionally associated with the HU in laying hens. The cytoskeleton is a key structure for maintaining cellular responses, and significant changes in this pathway suggest that the responsiveness and signal transduction in the magnum may have undergone substantial alterations.

### 2.3. scRNA-Seq Uncovers Epithelial and Plasma Cell Contributions to HU

**Figure 3:**
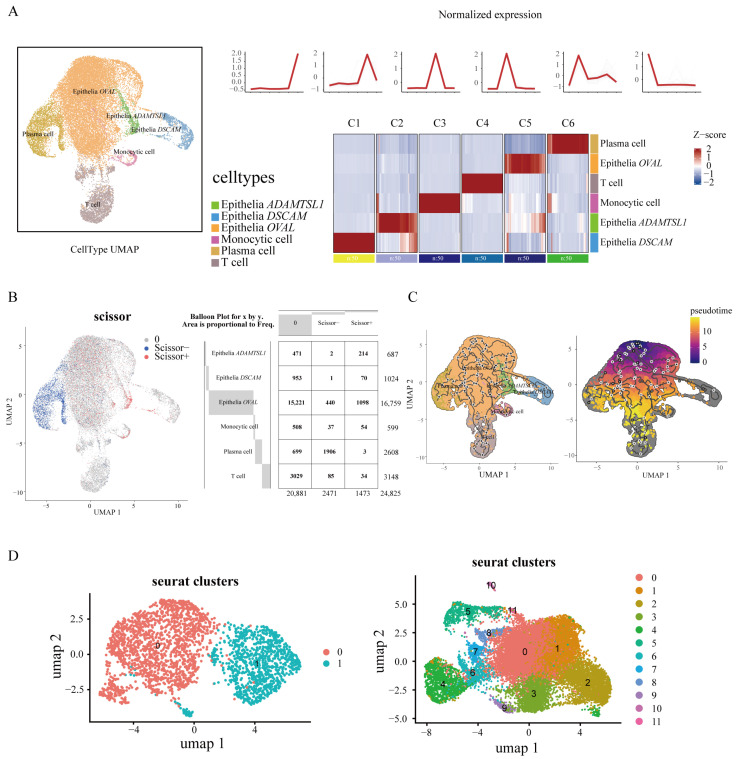
Single-cell transcriptomics reveals phenotype-associated epithelial subclusters and immune cell populations. (**A**) UMAP and clustergvis analysis of single-cell data. The left panel shows the UMAP plot and the right panel displays clustergvis results, including Mfuzz clustering and heatmaps of the top 50 genes per cell subcluster. Epithelial cells were classified as green (*ADAMTSL1*-high), blue (*DSCAM*-high), and orange (*OVAL*-high). Other cell types include purple (monocytic cells), mustard yellow (plasma cells), and brown (T cells). (**B**) SCISSOR analysis results. Blue denotes phenotype negatively correlated cell types, red indicates phenotype positively correlated cell types, and gray represents non-significant cell types. The gray boxes on the right show the proportion of predicted non-significant, negatively correlated, and positively correlated cells in different cell types. (**C**) Pseudotime trajectory reconstruction using Monocle3 (v1.3.7) and trajectory analysis with *OVAL*-high epithelial cells as the starting state. (**D**) Subcluster analysis of plasma cells and *ADAMTSL1*-/*OVAL*-high epithelial cells. Left panel shows subcluster analysis of plasma cells; right panel displays subcluster analysis of *ADAMTSL1*-/*OVAL*-high epithelial cells.

To investigate cellular changes associated with HU during tissue degeneration, we performed single-cell RNA sequencing (scRNA-seq) on the magnum to investigate the cellular changes associated with the HU during tissue degeneration. We identified six distinct cell populations, including three epithelial subtypes characterized by the high expression of *OVAL*, *ADAMTSL1*, and *DSCAM*, respectively, along with plasma cells, monocytes, and T cells (Figure 3A). The visualization of the top 50 highly expressed genes within each cell type revealed distinct expression patterns, supporting the robustness of our cell type classification and its suitability for downstream analysis (Figure 3A).

We integrated 12 genes identified from eQTL analysis with 259 genes derived from transcriptomic analysis and assessed the average expression and expression proportion of these genes across the six cell types. The results showed no obvious differences in either average expression levels or expression proportions among the six cell types (Figure S2A). Next, we performed Scissor analysis by integrating the single-cell transcriptomes with bulk RNA-seq expression data from the top five and bottom five individuals based on the HU, along with corresponding phenotype files. This analysis revealed that approximately 73% of plasma cells were negatively correlated with the HU, while about 30% of the *ADAMTSL1*-expressing epithelial cells showed a positive correlation (Figure 3B).

We performed pseudotemporal trajectory analysis using Monocle3 (v1.3.7) to investigate the developmental relationships among distinct cell types (Figure 3C). Notably, plasma cells exhibited the highest UMI counts, consistent with their antibody-secreting function, while *OVAL*-high epithelial cells showed the lowest UMI levels (Figure S2B). This observation suggested that *OVAL*-high epithelial cells may represent an early or precursor state in the differentiation hierarchy. Based on this, we manually selected the *OVAL*-high epithelial cells as the root in the Monocle3 (v1.3.7) analysis and observed a differentiation trajectory connecting these cells to the *ADAMTSL1*-high epithelial cells that are associated with the HU phenotype (Figure 3C).

### 2.4. Subclustering of Plasma and Epithelial Cells Reveal Key Genes

We further subclustered plasma cells and *ADAMTSL1*-/*OVAL*-high epithelial cells to identify finer-grained, phenotype-associated subpopulations for downstream analysis. We performed unsupervised clustering on plasma cells using a resolution parameter of 0.1, which identified two distinct subclusters (ego0–ego1; Figure 3D, Table S5). Gene Ontology (GO) enrichment analysis was then conducted for each set of marker genes to confirm their functional relevance. Subclusters ego0 and 1 were significantly enriched for GO terms, with functions predominantly related to immunity, ribosomes, and the peptide biosynthetic process (Figure S2C).

Similarly, we conducted subclustering analysis (resolution = 0.6) on the epithelial cell population characterized by the high expression of *ADAMTSL1* and *OVAL*, yielding twelve subclusters (ego0–ego11; Figure 3D, Table S6). GO analysis demonstrated that these epithelial subclusters were predominantly enriched for functions involving ribosomal activity, amide biosynthesis, and protein metabolism (Figure S2D).

To identify cell type-specific functions associated with genes related to the HU, we conducted Venn diagram analyses by integrating the 12 genes identified from the eQTL analysis and the 259 genes from the transcriptomic analysis with marker genes derived from plasma cells and epithelial subclusters with high *ADAMTSL1* or *OVAL* expression. The results revealed that plasma cell subcluster ego1 and epithelial subclusters ego4 and ego5 were functionally associated with these gene sets (Figure S2E). Subsequently, we applied CytoHubba to visualize hub genes within these subclusters. The analysis indicated that ribosome-related functions in plasma cells, as well as ribosomal and cytoskeletal functions in epithelial cells, were closely associated with the HU during the late stage of egg production (Figure S2F).

### 2.5. Integrative Multi-Omics Analysis Identifies Key Genetic Regulators of HU

To identify genes whose expression levels are associated with the HU, we performed a TWAS analysis using SPrediXcan, selecting genes with a significance threshold of *p*-value < 0.05. Subsequently, we integrated 12 candidate genes identified from eQTL analysis with 259 genes derived from transcriptomic profiling and intersected these with the TWAS-significant genes. This approach yielded a final set of 19 genes significantly associated with the HU at 66, 72, 80, 90, and 100 weeks of age (Table 1). Notably, three genes, *CISD1*, *NQO2*, and *SLC22A23*, were found to overlap with genomic regions containing significant eQTL variants, suggesting potential regulatory relationships between these loci and gene expression. To further elucidate the functional impact of these genes on the HU and validate the robustness of our analyses, we conducted a SMR analysis using eQTL summary statistics. The SMR results confirmed that *NQO2*, *VRK2*, and *TMEM130* exhibited significant associations with the HU (*p*_SMR < 0.05). Importantly, *VRK2* and *TMEM130* were consistently identified in both TWAS and transcriptomic analyses, underscoring the high concordance between these independent approaches and reinforcing the credibility of our findings.

**Figure 4:**
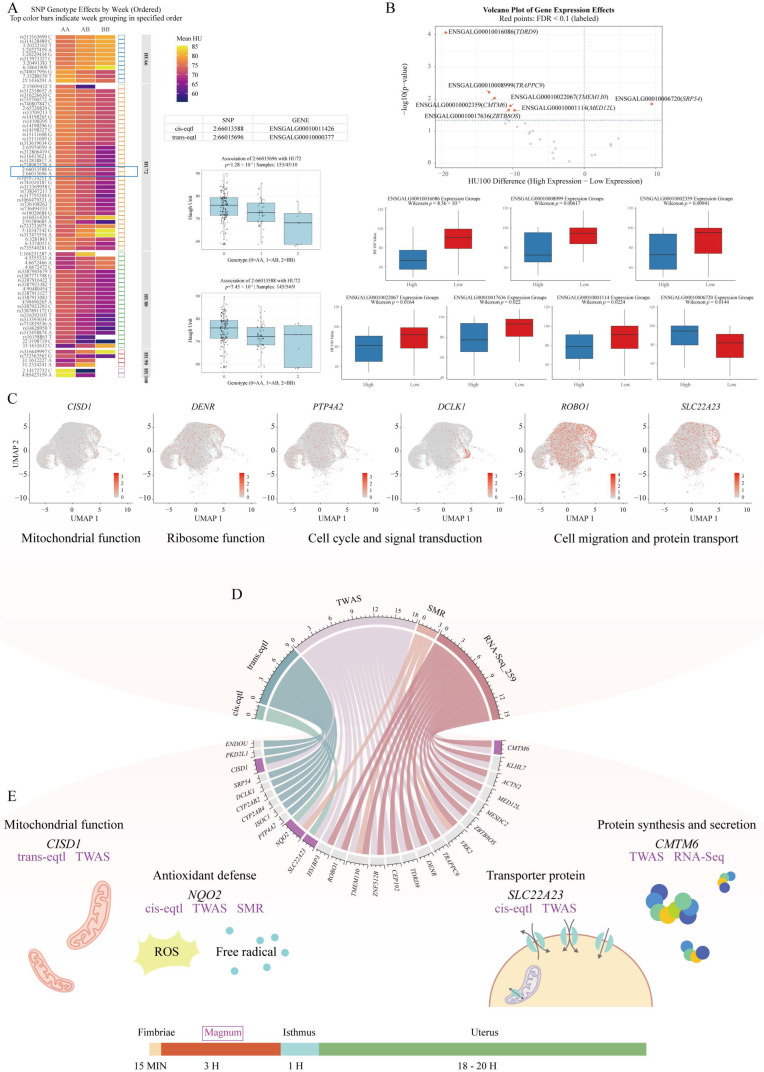
Integrative multi-omics analysis identifies genetic and molecular regulators of Haugh unit (HU) phenotype. (**A**) Effects of SNP genotypes on HU values. Genotypes are coded as 0 = AA (homozygous reference), 1 = AB (heterozygous), and 2 = BB (homozygous alternative). The SNP marked by blue boxes are shown in detail in the accompanying table and boxplot to the right. (**B**) HU phenotype differences between high- and low-expression groups for candidate genes. (**C**) Cell-type-specific expression patterns of HU-associated genes. Red indicates positive expression of target genes; gray indicates no detectable expression. (**D**) Consensus evidence from four analytical approaches (eQTL mapping, TWAS, SMR, and transcriptomics) for the 28 candidate genes. (**E**) Regulatory patterns of four key genes *CISD1*, *NQO2*, *SLC22A23*, and *CMTM6*.

The subsequent genotyping analysis of significant SNP loci from GWAS revealed that the increasing mutation load at most loci was associated with a progressive decline in the HU (Figure 4A). Notably, two eQTL-linked loci (2:66013588 and 2:66015696), which regulate the expression of ENSGALG00010011426 (*NQO2*) and ENSGALG00010000377, demonstrated pronounced effects. While the majority of individuals exhibited wild-type genotypes (0 = AA), homozygous mutant genotypes (2 = BB) showed elevated phenotypic heterogeneity in HU measurements, suggesting detrimental impacts of biallelic mutations on the albumen quality.

Through integrative analysis combining eQTL, TWAS, SMR, and transcriptomic data, we identified 28 candidate genes. Using single-cell transcriptomic data, we examined the cellular localization of these 28 genes. The results showed that *CMTM6*, *KLHL7*, *MESDC2*, *VRK2*, *DENR*, *CEP192*, *ROBO1*, *SLC22A23*, *PTP4A2*, *SRP54*, and *CISD1* were highly expressed in *OVAL*-high epithelial cells (Figure S3A). Expression-based stratification (top/bottom 25 samples) revealed seven genes (*TDRD9*, *TRAPPC9*, *CMTM6*, *ZBTB8OS*, *TMEM130*, *MED12L*, and *SRP54*) with significant HU phenotype differences (FDR-adjusted *p* < 0.1; Figure 4B). Notably, the elevated expression of *TMEM130*, *ZBTB8OS*, and *MED12L* correlated with stable HU maintenance, whereas the high expression of *TRAPPC9* and *CMTM6* was associated with increased HU variability. Cell-type-specific expression profiling demonstrated that mitochondrial ribosome-associated genes (*CISD1*, *DENR*) and signaling regulators (*PTP4A2*) were ubiquitously expressed across plasma cells and *ADAMTSL1*-/*OVAL*-high epithelial cells. In contrast, *DCLK1*, a cell cycle and signaling modulator, showed preferential expression in *ADAMTSL1*-high epithelial populations, suggesting its specialized regulatory role in this subset (Figure 4C). Furthermore, cytoskeletal genes (*ROBO1*, *SLC22A23*) were enriched in *OVAL*-high epithelial cells, implicating structural organization and secretory machinery as critical determinants of the HU.

Following a comprehensive assessment of 28 candidate genes, we proposed four genes as strong candidates for HU regulation based on the genomic location and multi-omics evidence (Figure 4E). *CISD1* is regulated by the SNP 2:66015696 and shows the HU association in TWAS. *NQO2* is influenced by the 65.91–66.03 mb on chromosome 2 and demonstrated consistent HU associations in both TWAS and SMR analyses. *SLC22A23*, regulated by variants in the 65.91–66.03 mb on chromosome 2, is associated with the HU according to the TWAS results. *CMTM6* showed consistent associations with the HU in both the TWAS and transcriptomic analyses. These four genes represent the most promising genetic determinants of the HU, with each showing consistent evidence from multiple independent analytical frameworks. Their identification through this integrative approach strongly suggests their functional importance in regulating albumen quality traits.

To further validate the association between the genes *CISD1*, *NQO2*, *SLC22A23*, and *CMTM6* and the HU, we divided the samples into high- and low-expression groups (n = 29) based on the expression levels of each gene. A t-test on HU values between these groups revealed significant associations for *NQO2* and *CMTM6* (*p* = 0.0442 and 0.0116, respectively) (Figure S3B). Meanwhile, to investigate the dynamic expression patterns of these genes during the decline in the poultry production performance, we visualized their trends using previously generated transcriptomic data from magnum tissues of Rhode Island Red hens at 50, 70, and 100 weeks of age. The results showed that the expression levels of all four core genes slightly decreased from 50 to 70 weeks, but exhibited a marked rebound at 100 weeks, surpassing their expression levels at 50 weeks (Figure S3C). This expression pattern further supports the potential role of these genes in regulating the albumen quality during the later stages of the laying cycle.

## 3. Discussion

The aging process in laying hens is invariably accompanied by a decline in the reproductive tract function, leading to a deteriorated egg quality and increased heterogeneity in production traits during the late laying phase [18]. The albumen quality is a critical determinant of the poultry production efficiency and consumer acceptance, with HU serving as a widely recognized metric for the albumen quality and freshness. The deterioration of the albumen quality, particularly during the terminal laying period, significantly compromises the economic efficiency of the poultry industry [19]. Consequently, improving the albumen quality in late-lay hens to extend productive cycles has become an urgent priority. In laying hens, the yolk resides in the magnum for roughly three hours, during which the protein concentration is twice that of the mature albumen, while the overall albumen volume is only half of its final volume [4]. This indicates that after it exits the magnum, protein secretion halts and subsequent oviduct regions largely add water to dilute the albumen. Therefore, alterations in the magnum function likely underlie the HU decline, especially in late-laying hens. This study employed an integrative multi-omics approach to elucidate the molecular mechanisms underlying the magnum-specific regulation of the HU during the late laying period.

Through genome-wide association analysis integrating genomic data with HU phenotypic measurements at 66, 72, 80, 90, and 100 weeks of age, we identified 77 significant SNPs associated with albumen quality traits. These findings demonstrate that the dynamic changes in the HU during the late laying period are polygenic in nature, being regulated by multiple loci distributed across the genome rather than being controlled by specific chromosomal regions. Meanwhile, our eQTL analysis revealed that the genes *NQO2* and ENSGALG00010011348 are both regulated by cis-eQTLs located within a highly overlapping region (65.91–66.03 Mb) on chromosome 2. In addition, several genes, including ENSGALG00010000377, *PTP4A2*, *ISOC1*, *CYP2AB4*, *CYP2AB2*, *DCLK1*, *SRP54*, *CISD1*, *PKD2L1*, and *ENDOU*, were identified as being regulated by trans-eQTLs. These genes participate in various biological processes including cellular signaling (*PTP4A2*, *PKD2L1*) [20,21], metabolic functions (*CYP2AB4*, *CYP2AB2*) [22,23], and nucleic acid metabolism (*ENDOU*, *SRP54*) [22,24,25]. The identification of these genes indicates the existence of more complex, network-based regulatory mechanisms influencing the albumen quality during the late laying period.

In the transcriptomic data, we employed two widely used machine learning methods, linear regression and random forest, to identify genes significantly associated with the HU. These methods have been successfully applied in poultry production research. Bermann M et al. demonstrated that linear regression models incorporating genomic data could significantly improve the prediction accuracy of broiler mortality rates [26]. Additionally, random forest models have been utilized to estimate the broiler body weight and identify candidate genes associated with chicken immune traits [27,28]. The KEGG enrichment analysis revealed significant pathway enrichment in several biological processes, including cytoskeletal organization in muscle cells, pyruvate metabolism, and folate biosynthesis. The cytoskeleton serves as a mechanotransduction network that propagates internal and external physical forces to modulate cell behavior [29]. Research demonstrated that age-related molecular functional changes are closely linked to cytoskeletal reorganization, where aging alters actin expression to modify actin cytoskeleton organization and dynamics [30,31,32]. This suggested that functional alterations in the cytoskeleton may be a key determinant underlying the increased variability in the HU during late-stage egg production. The co-enrichment of mitochondrial-related pathways (pyruvate metabolism/folate biosynthesis) is particularly noteworthy, as impaired mitochondrial energetics are known to (1) accelerate cellular senescence [33] and (2) reduce the biosynthetic capacity [34].

In the scRNA-Seq analysis, we identified *ADAMTSL1*-/*OVAL*-high epithelial cells and plasma cells as key contributors to functional changes in the magnum during the late laying stage. These findings are further supported by the morphological characteristics observed in the magnum at 100 weeks of age, including incomplete epithelial folding, a loosened cellular network, and a marked reduction in mucosal folds [15]. It has been observed that in aged laying hens, a large number of immune-related genes exhibit a downregulated expression trend [35]. Comparative analysis between young and aged hens has revealed significant alterations in immunoglobulins, which play a key role in the transmission of immunity [36]. This is consistent with our findings that changes in the plasma cell function, which are closely associated with immune function, may influence the egg quality during the later stages of the laying period. Notably, genes associated with ribosomal function and cytoskeletal organization were further localized to these cell populations. These findings are consistent with the results from transcriptomic analysis, reinforcing their potential role in regulating the albumen quality. Some studies have suggested that the presence of an egg in the magnum induces the mechanical distention of the magnum wall, which in turn stimulates the secretion of stored egg-white proteins [37], potentially mediated by the mechanotransductive function of the cytoskeleton.

To integrate transcriptomic and genomic data, we performed TWAS and SMR analyses to identify genes associated with the HU phenotype. TWAS combine GWAS summary statistics with gene expression data to prioritize genes whose genetically predicted expression is associated with the HU [38]. SMR is a powerful approach that leverages gene expression and phenotype summary data to infer potential causal relationships between the two, thereby reducing confounding effects due to linkage. Nineteen genes identified in the TWAS analysis overlapped with those from previous analyses, further highlighting their potential functional roles in the regulation of the HU during the late laying period. Among the SMR results, *NQO2*, *VRK2*, and *TMEM130* were suggested to have a causal relationship with the HU and are known to play important roles in antioxidant defense [39], apoptosis [40], and cell migration [41], respectively.

Based on integrated analyses of eQTL, TWAS, and SMR results, we identified *CISD1*, *NQO2*, *SLC22A23*, and *CMTM6* as robustly associated with the HU. *CISD1* is localized to the outer mitochondrial membrane and functions in modulating the mitochondrial oxidative capacity and cellular energy metabolism [42]. Studies suggested this gene may be associated with egg production traits and related phenotypes in poultry species [43]. *NQO2* is a flavoprotein that plays a crucial role in cellular protection against oxidative stress [39]. Solute carriers (*SLCs*) are a family of highly specific membrane transport proteins responsible for the translocation of various solutes, including amino acids, organic and inorganic ions, and sugars. Evidence suggests that *SLC* family genes actively participate in the transport of precursor molecules, thereby playing a critical role in the synthesis of albumen proteins [44]. In human studies, *SLC* transporters have been identified as important targets in biopharmaceutical development, highlighting the potential for targeting *SLC* genes to regulate the production performance during the late laying stage in poultry [45,46,47]. Previous studies have shown that genes such as *CMTM6* play regulatory roles in protein synthesis and secretion processes [48].

These findings highlight critical cellular processes that underpin the albumen quality, offering novel molecular targets to improve the egg production stability in aging hens through potential genetic selection and nutritional interventions. Our approach also offers a framework for future studies on host–microbiota interactions and gene function validation.

## 4. Materials and Methods

### 4.1. Ethics Statement

All experimental procedures, including tissue collection and phenotypic observations, were conducted in accordance with the guidelines and regulations of the Institutional Animal Care and Use Committee (IACUC) at China Agricultural University (Permit Number: AW21214202-1-1). The protocols were reviewed and approved by the IACUC before the commencement of this study.

### 4.2. Experiment Animals and Sample Collection

A total of 254 Rhode Island Red laying hens were obtained from Beijing Huadu Yukou Poultry Co. Ltd. (Beijing, China) and housed individually under standardized husbandry conditions until 100 weeks of age. HU values were monitored throughout the laying period. Eggs were collected continuously for one week at 66, 72, 80, 90, and 100 weeks of age, and the HU was measured to assess the egg albumen quality.

All 254 hens were euthanized and magnum tissues were harvested for whole-genome sequencing and RNA sequencing at 100 weeks of age, enabling integrative multi-omics analyses. Additionally, we selected representative magnum tissue actively secreting egg white for scRNA-seq analysis.

### 4.3. RNA Extraction, Quality Evaluation, and Sequencing

Total RNA was extracted and purified from the samples using TRIzol reagent (Thermo Fisher Scientific, Waltham, MA, USA), following the manufacturer’s instructions. The quality and integrity of the RNA were then evaluated to ensure suitability for downstream applications. After purification, PCR amplification was carried out. The resulting libraries were sequenced using the Illumina NovaSeq 6000 platform in paired-end mode (PE150), following standard protocols.

### 4.4. Transcriptome Data Processing

- Quality control. The raw sequencing data underwent quality control processing to eliminate low-quality reads using Cutadapt software (v1.9), resulting in clean data [49,50].
- Reads alignment. Reads from all samples were aligned to the chicken reference genome (Gallus gallus, GCA_016699485.1, https://mart.ensembl.org/Gallus_gallus/Info/Annotation# URL (accessed on 1 November 2024)) using HISAT2 (v 2.2.1). Prior to the alignment, low-quality reads were filtered based on the quality scores associated with each read. HISAT2 allows multiple alignments per read (up to 20 by default) and permits up to two mismatches during alignment. Additionally, HISAT2 constructs a database of potential splice junctions, enabling the mapping of reads that initially failed to align by comparing them against this junction database [51,52].
- Transcript Assembly. Transcript abundance was estimated using StringTie (v 2.1.6) in combination with Ballgown. Gene and mRNA expression levels were quantified based on FPKM (Fragments Per Kilobase of transcript per Million mapped reads), which normalizes transcript counts for both the sequencing depth and transcript length [53,54,55].

### 4.5. Single-Cell RNA-Seq Sample Preparation and Data Analysis

Tissue samples were surgically collected and stored in MACS Tissue Storage Solution (Miltenyi Biotec, Bergisch Gladbach, Germany) prior to processing. Samples were washed with PBS, minced into ~1 mm^3^ pieces on ice, and digested with 1 mg/mL collagenase IV and 30 U/mL DNase I (Worthington) at 37 °C for two 10 min rounds. The resulting cell suspension was filtered through a 70 µm strainer, centrifuged at 300 g for 5 min, and treated with red blood cell lysis buffer (Miltenyi Biotec) for 8 min. After washing and resuspension in PBS with 0.04% BSA, the cells were passed through a 35 µm strainer. Cell viability was assessed using AO/PI staining and a Countstar Fluorescence Cell Analyzer. Live cells were enriched using the MACS Dead Cell Removal Kit (Miltenyi Biotec).

scRNA-seq data analysis was performed using the NovelBrain Cloud Analysis Platform (www.novelbrain.com URL (accessed on 20 November 2024)). Raw reads were processed with fastp to remove adapters and low-quality reads, and aligned to the chicken genome (GRCg7b Ensembl108) using CellRanger v7.1.0 to generate feature–barcode matrices. Samples were downsampled based on mapped barcoded reads per cell to obtain a unified expression matrix. DoubletFinder (v 2.0) was used to remove potential doublets and low-quality cells were excluded based on the following criteria: fewer than 200 or more than 6000 detected genes, >20% mitochondrial or red blood cell gene UMI content, and genes detected in fewer than 3 cells. Data were normalized and scaled using the Seurat package (v4.0.3), regressing out total UMI counts and mitochondrial content. Batch effects were corrected using the MNN (mutual nearest neighbor). Principal component analysis (PCA) was conducted using the top 2000 highly variable genes, and the top 10 PCs were used for tSNE and UMAP construction. Clustering was performed using a graph-based method and marker genes were identified with the FindAllMarkers function (Wilcoxon rank-sum test, logFC > 0.25, *p* < 0.05, min.pct > 0.1). To further define cell types, clusters of the same lineage were subjected to additional re-tSNE analysis, clustering, and marker identification.

### 4.6. GWAS

The GWAS was performed using GCTA (v1.94.0) with a fastGWA-mixed linear model (fastGWA-mlm). First, a genetic relationship matrix (GRM) was constructed using quality-controlled autosomal SNPs (autosome-num 39) to estimate pairwise genetic similarities between individuals. To improve the computational efficiency, the dense GRM was converted into a sparse GRM (threshold = 0.05). The top 10 principal components (PCs), derived from the GRM, were included as covariates to correct for population stratification.

### 4.7. Linkage Disequilibrium (LD) Analysis

To further explore the genomic context of significant GWAS loci, we conducted regional LD analysis. Representative GWAS peaks were selected for LD mapping based on Manhattan plots. LD blocks were visualized using LDBlockShow, which generates LD heatmaps based on pairwise r^2^ values calculated from SNP genotypes. SNPs within each haplotype block were defined using the software’s default parameters. The initial significant SNPs were expanded by including additional variants located within the same LD blocks, yielding a broader candidate SNP set for downstream analyses such as eQTL mapping and gene prioritization.

### 4.8. Expression Quantitative Trait Loci (eQTL) Analysis

eQTL analysis was performed using the Matrix eQTL to identify genetic variants associated with the transcript abundance. The analysis was conducted on a Linux-based server environment using Xshell 8 (Build 0084) as the terminal interface. A linear regression model (modelLINEAR) was employed and no covariates were included due to the absence of known confounding factors.

Both cis- and trans-eQTL analyses were performed. Cis-eQTLs were defined as SNPs located within ±1 Mb of the transcription start site of a gene. The significance thresholds were set to 2 × 10^−5^ for cis-eQTLs and 1 × 10^−5^ for trans-eQTLs. Default settings were used for file slicing and memory management. No missing genotype imputation or expression filtering was performed, and the analysis included only autosomal variants. After computation, Q–Q plots were generated to assess the distribution of *p*-values. False discovery rate (FDR) correction was applied using the Benjamini–Hochberg method and significant eQTLs were filtered at FDR < 0.05.

### 4.9. Transcriptome-Wide Association Study (TWAS) Analysis

To investigate the genetic regulation of the HU through gene expression mechanisms, we performed a TWAS using the S-PrediXcan (v 1.0) framework implemented in the MetaXcan (v 0.7.3) software suite. This approach integrates genotype data with transcriptomic prediction models to identify genes whose expression levels are significantly associated with the target phenotype.

### 4.10. Summary-Based Mendelian Randomization (SMR) Analysis

To investigate potential causal relationships between gene expression and the HU phenotype, we performed Mendelian randomization analysis using the SMR software (v 1.3.2), which integrates summary-level data from GWAS and eQTL studies. eQTL summary data generated from cis-eQTL analysis were converted into the BESD format using the SMR utility. This approach uses genetic variants as instrumental variables to assess the pleiotropic or causal effects of gene expression on the HU trait.

### 4.11. SCISSOR Analysis

Scissor (v 2.0.0) was employed to integrate bulk RNA-seq data with single-cell transcriptomic profiles from the magnum of Rhode Island Red hens [56]. Bulk expression matrices and corresponding phenotype data—specifically HU values and HU-based groupings (Low vs. High)—were preprocessed to remove missing values and ensure matched sample identifiers. To emphasize phenotypic extremes, Scissor analysis was performed using bulk samples derived from the top 5 and bottom 5 hens ranked by HU values at 100 weeks of age. The single-cell dataset was formatted as a Seurat object. Scissor was run using the group label (“status”) as the response variable to identify cell populations associated with egg quality traits. A grid search was conducted to determine the optimal alpha parameter, and a cutoff threshold was applied to identify Scissor^+^ and Scissor^−^ cells.

### 4.12. Monocle3-Based Pseudotime Analysis

To reconstruct cellular trajectories and infer pseudotime dynamics, we utilized the Monocle3 (v 1.3.7) R package. The gene expression count matrix, cell-level metadata, and gene annotations were extracted from the processed Seurat object and used to construct a CellDataSet object compatible with Monocle3. To preserve consistency in the visualization of cell states across analysis platforms, the UMAP embeddings obtained from Seurat were transferred and manually assigned to the Monocle3 object, ensuring alignment with previous visual representations.

### 4.13. Cell Subcluster Analysis

To further characterize the heterogeneity within a specific cell population, we isolated the subset of interest from the single-cell dataset. Clusters were determined using the FindClusters function in Seurat. A new Seurat object was reconstructed using the raw count matrix and corresponding metadata, and principal component analysis (PCA) was re-performed following standard preprocessing steps, including normalization, the identification of highly variable genes, and data scaling. To ensure consistency across analyses, previously computed PCA embeddings were retained by manually assigning them to the PCA slot of the Seurat object.

The target cell population was extracted based on its annotated cell type label and downstream analyses were conducted on this subset. After re-normalization and dimensionality reduction via PCA, Uniform Manifold Approximation and Projection (UMAP) was applied to visualize cell states in a low-dimensional space. A shared nearest neighbor (SNN) graph was constructed using the top 10 principal components, followed by unsupervised clustering at a resolution of 0.1 and 0.6 to identify distinct subpopulations.

To identify cluster-specific marker genes, we applied the FindAllMarkers function in Seurat using the default Wilcoxon rank sum test. Only positively enriched markers were considered (only.pos = True) and filtering thresholds were set to retain genes expressed in at least 50% of cells within a given cluster (min.pct = 0.5). The log fold-change threshold (logfc.threshold) was adjusted based on dataset-specific characteristics to ensure the selection of biologically meaningful markers.

### 4.14. Enrichment Analysis

Functional enrichment analysis was performed to explore the biological pathways associated with aging-related differentially expressed genes (DEGs). The Gene Ontology (GO) database (http://www.geneontology.org/ URL (accessed on 20 March 2025)) classifies gene functions into three categories: the biological process, molecular function, and cellular component [57]. The Kyoto Encyclopedia of Genes and Genomes (KEGG) database (http://www.genome.jp/kegg/ URL (accessed on 20 March 2025) offers a comprehensive view of biological systems from functional, genomic, and chemical perspectives [58]. Enrichment analysis was conducted using the clusterProfiler package (v4.6.2), with a significance threshold set at *q* < 0.05 [59,60].

### 4.15. Linear Regression and Random Forest Analysis

To identify genes exhibiting expression trends across phenotypic groups, a linear regression model was applied to the gene expression matrix using the broom (v1.0.7) R package, which facilitated the tidy extraction of regression coefficients and *p*-values associated with phenotype variation. Specifically, the phenotype groups (“Low,” “Medium,” and “High”) were first encoded as an ordered numeric variable. For each gene, a linear model was fitted with the gene’s expression values as the response variable and the numeric group label as the predictor. Genes with a nominal *p*-value < 0.05 and an absolute effect size above the 90th percentile were considered to exhibit significant expression trends across phenotypic groups.

In parallel, a random forest (RF) classifier was implemented using the randomForest (v4.7-1.2) R package. In this analysis, gene expression data were transposed so that genes served as predictors and samples as observations, with the phenotype group labels used as the categorical response variable. The RF model was trained using default parameters and a fixed random seed to ensure reproducibility. Variable importance was evaluated using the Mean Decrease Accuracy (MDA) metric, and the top 10% of genes ranked by importance were defined as key contributors to the phenotype classification.

### 4.16. PPI Network Construction and Network Integration

A protein–protein interaction (PPI) network was constructed using the STRING database (Search Tool for the Retrieval of Interacting Genes/Proteins) [61]. Interactions with a combined score greater than 0.4, indicating medium or higher confidence, were selected to build the PPI network using Cytoscape software (v 3.9.1) [62]. To identify hub genes within the network, the CytoHubba plugin in Cytoscape was employed, applying multiple topological algorithms and centrality measures based on the shortest path analysis [63]. The top 10 ranked nodes were identified using the Neighborhood Connectivity Centrality (NCC) method. These hub genes represent core proteins that may function as key regulators with essential biological roles.

## 5. Conclusions

Through integrative HU phenotypes from five timepoints (66, 72, 80, 90, and 100 weeks of age) with genomic, RNA-Seq, and scRNA-Seq data, we identified 77 significant SNPs associated with HU. scRNA-Seq identified plasma cells and *ADAMTSL1*-/*OVAL*-high epithelial cells as key cellular regulators of the HU. Furthermore, critical genes including *CISD1* (mitochondrial function), *NQO2* (antioxidant defense), *SLC22A23* (membrane transport), and *CMTM6* (protein synthesis and secretion) were found to be significantly associated with the HU. These findings provide novel insights into the molecular and cellular mechanisms underlying albumen quality deterioration during prolonged laying cycles.

## Figures and Tables

**Table 1:** The results of TWAS.

Trait	Gene	Gene Symbol	Zscore	Effect Size	*p* Value
HU66	ENSGALG00010009119	*CISD1*	−3.364072985	−7.744721041	0.000768012
	ENSGALG00010002359	*CMTM6*	2.334779743	3.195354674	0.01955493
	ENSGALG00010014250	*-*	2.246253145	5.884450865	0.024687798
	ENSGALG00010001397	*KLHL7*	−2.081680939	−2.798993813	0.037371623
	ENSGALG00010009118	*ACTN2*	2.027599228	5.276012722	0.042601168
	ENSGALG00010001114	*MED12L*	−2.005956946	−2.705734531	0.044860842
	ENSGALG00010019300	*MESDC2*	−1.990618634	−4.600845455	0.04652283
	ENSGALG00010017636	*ZBTB8OS*	−1.968763637	−2.268956978	0.04898024
HU72	ENSGALG00010009119	*CISD1*	−3.64988896	−8.863673597	0.000262354
	ENSGALG00010011426	*NQO2*	−3.332953216	−3.121989042	0.000859294
	ENSGALG00010017065	*VRK2*	2.480160034	2.156703244	0.013132343
	ENSGALG00010008999	*TRAPPC9*	−2.468575317	−6.050582223	0.01356521
	ENSGALG00010011348	*SLC22A23*	2.189596504	4.01736676	0.028553513
HU80	ENSGALG00010008999	*TRAPPC9*	−2.679306244	−7.693929247	0.007377489
	ENSGALG00010009118	*ACTN2*	2.457223144	7.795617395	0.014001569
	ENSGALG00010025026	*DENR*	−2.124010969	−3.587129831	0.033669226
	ENSGALG00010016086	*TDRD9*	1.990443239	4.307971134	0.046542131
	ENSGALG00010001205	*CEP192*	−1.963226047	−2.906827479	0.049619914
HU90	ENSGALG00010001205	*CEP192*	−2.316489697	−4.126937371	0.020531546
	ENSGALG00010022578	*ZNF512B*	2.024957407	4.407960469	0.042871738
HU100	ENSGALG00010016086	*TDRD9*	−2.393592171	−12.11901395	0.016684289
	ENSGALG00010022067	*TMEM130*	−2.277763246	−6.529320887	0.022740687
	ENSGALG00010007360	*ROBO1*	−2.19131752	−6.630930423	0.028428823
	ENSGALG00010007956	*HS1BP3*	−1.969423368	−13.89364606	0.048904495
	ENSGALG00010017065	*VRK2*	−1.9624531	−4.634019521	0.049709756

TWAS: Transcriptome-wide association study analysis; HU: Haugh unit.

## Data Availability

The original contributions presented in this study are included in the article/Supplementary Materials. Further inquiries can be directed to the corresponding author.

## Supplementary Materials

The following supporting information can be downloaded at: https://www.mdpi.com/article/10.3390/ijms26167876/s1.

## Abbreviations

The following abbreviations are used in this manuscript:

HU	Haugh unit
GWAS	Genome-wide association studies
eQTL mapping	Expression quantitative trait locus mapping
TWAS	Transcriptome-Wide Association Study
LD	Linear dichroism
SMR analyses	Summary-data-based Mendelian Randomization analyses
SNPs	single-nucleotide polymorphisms
scRNA-seq	single-cell RNA sequencing
KEGG	Kyoto Encyclopedia of Genes and Genomes
GO	Gene Ontology
DEGs	differentially expressed genes
PPI	protein–protein interaction
*CISD1*	CDGSH Iron–Sulfur Domain-Containing Protein 1
*NQO2*	quinone oxidoreductase 2
*SLCs*	Solute carriers
*CMTM6*	CKLF-like MARVEL transmembrane domain-containing family member 6
