# Supplementary material for: Multi-Omics Reveals Molecular and Genetic Mechanisms Underlying Egg Albumen Quality Decline in Aging Laying Hens
Source: Int J Mol Sci. 2025 Aug 15;26(16):7876. doi: 10.3390/ijms26167876 (PMC12386583; doi:10.3390/ijms26167876)
Supplement: Supplementary file 1 [file ijms-26-07876-s001.zip › ijms-3785126-supplementary-final.pdf]

Supplementary Tables

Table S1. Results of GWAS analysis
Table S2. Candidate SNPs Within LD Blocks of Significant Variants
Table S3. Results of cis-eQTL and trans-eQTL analysis
Table S4. Results of linear regression and random forest analysis
Table S5. Marker genes of plasma cell subpopulation
Table S6. Marker genes of *ADAMTSL1*-/*OVAL*-high epithelial subpopulation

Supplementary Figures

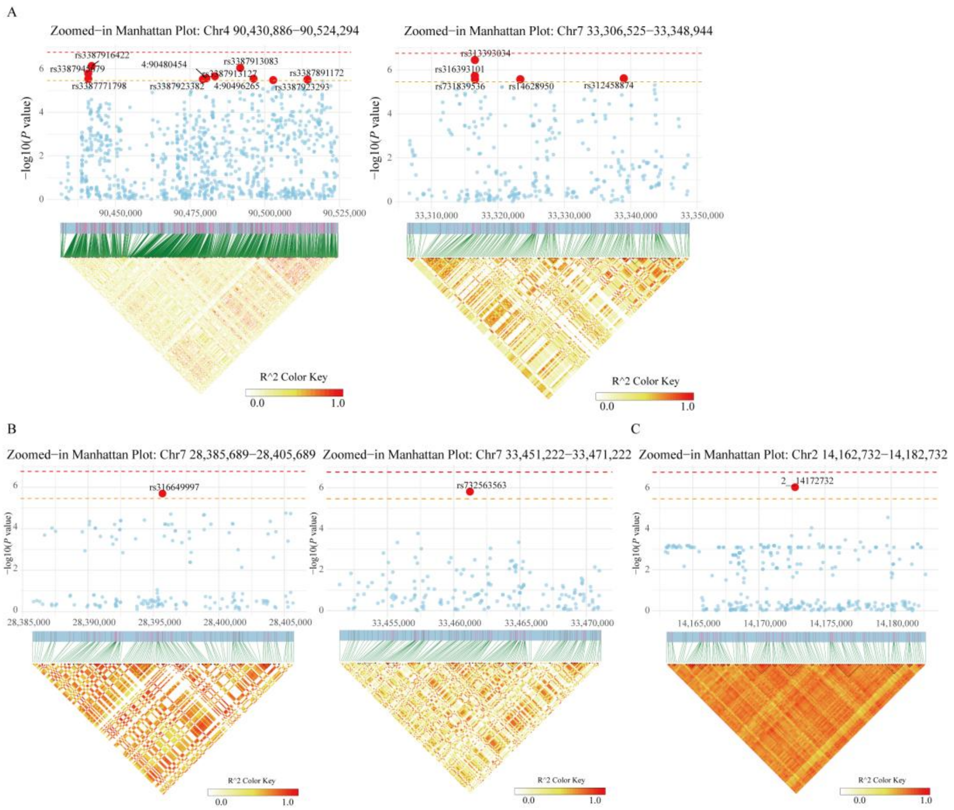

Figure S1. Regional association and linkage disequilibrium (LD) analysis of significant Haugh unit (HU)-associated loci. (A-C) Regional plots of top GWAS signals for HU at 80 (A), 90 (B) and 100 (C) weeks of age. Each panel includes a Manhattan plot (upper) and the corresponding linkage disequilibrium (LD) heatmap (lower). In the Manhattan plots, two dashed horizontal lines indicate the genome-wide significance threshold ($p < 1.74 \times 10^{-7}$) and the suggestive significance threshold ($p < 3.48 \times 10^{-6}$), with red dots indicating significant loci. In the LD heatmaps, color intensity reflects the strength of pairwise linkage disequilibrium, with red indicating stronger LD.

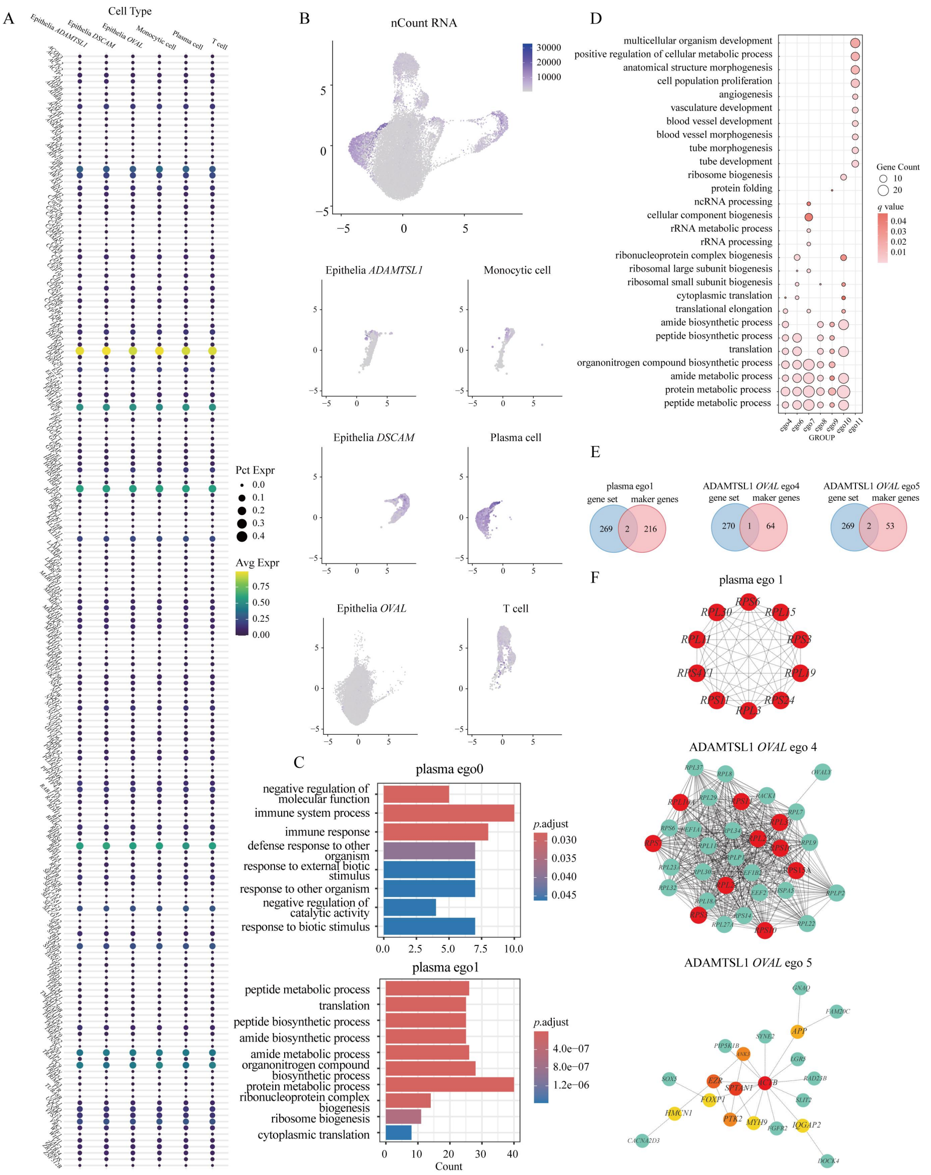

Figure S2. Characterization of candidate gene expression patterns and functional enrichment across cell populations. (A) Expression profiles of candidate genes in six cell types. Color intensity represents mean expression level, while dot size indicates expression proportion. (B) RNA content distribution across cell populations. (C) GO enrichment analysis of marker genes in plasma cell subclusters. (D) Functional annotation of marker genes in *ADAMTSL1*-/*OVAL*-high epithelial subclusters (bubble plot: circle size = gene count; color = -log10(*p* value)). (E) Venn diagram analysis of overlapping genes. (F) CytoHubba network analysis identifying top 10 hub genes (red-orange gradient) in plasma cells and *ADAMTSL1*-/*OVAL*-high epithelial subclusters.

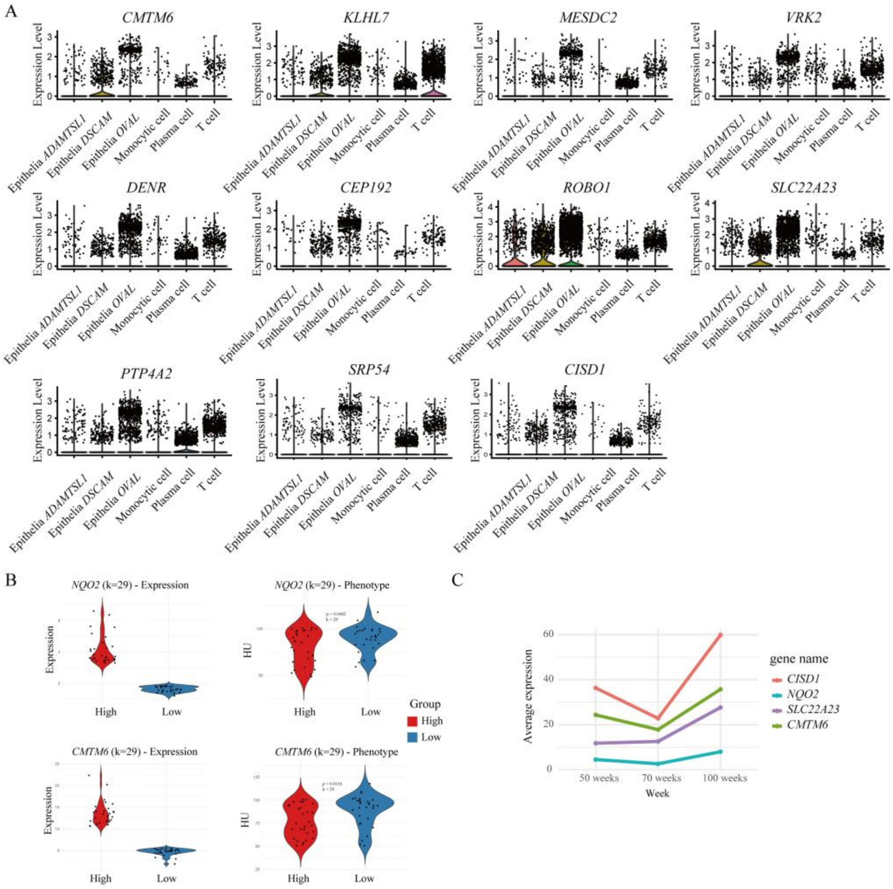

Figure S3. Expression patterns of key genes associated with albumen quality. (A) Violin plots showing genes highly expressed in *OVAL*-high epithelial cells. (B) Violin plots of *NQO2* and *CMTM6* showing gene expression levels (left) and corresponding HU phenotypes (right). (C) Expression levels of *CISD1*, *NQO2*, *SLC22A23*, and *CMTM6* in magnum tissue at 50, 70, and 100 weeks of age.
