# Supplementary figures and images for: Multi-Omics Reveals Molecular and Genetic Mechanisms Underlying Egg Albumen Quality Decline in Aging Laying Hens
Source: Int J Mol Sci. 2025 Aug 15;26(16):7876. doi: 10.3390/ijms26167876 (PMC12386583; doi:10.3390/ijms26167876)

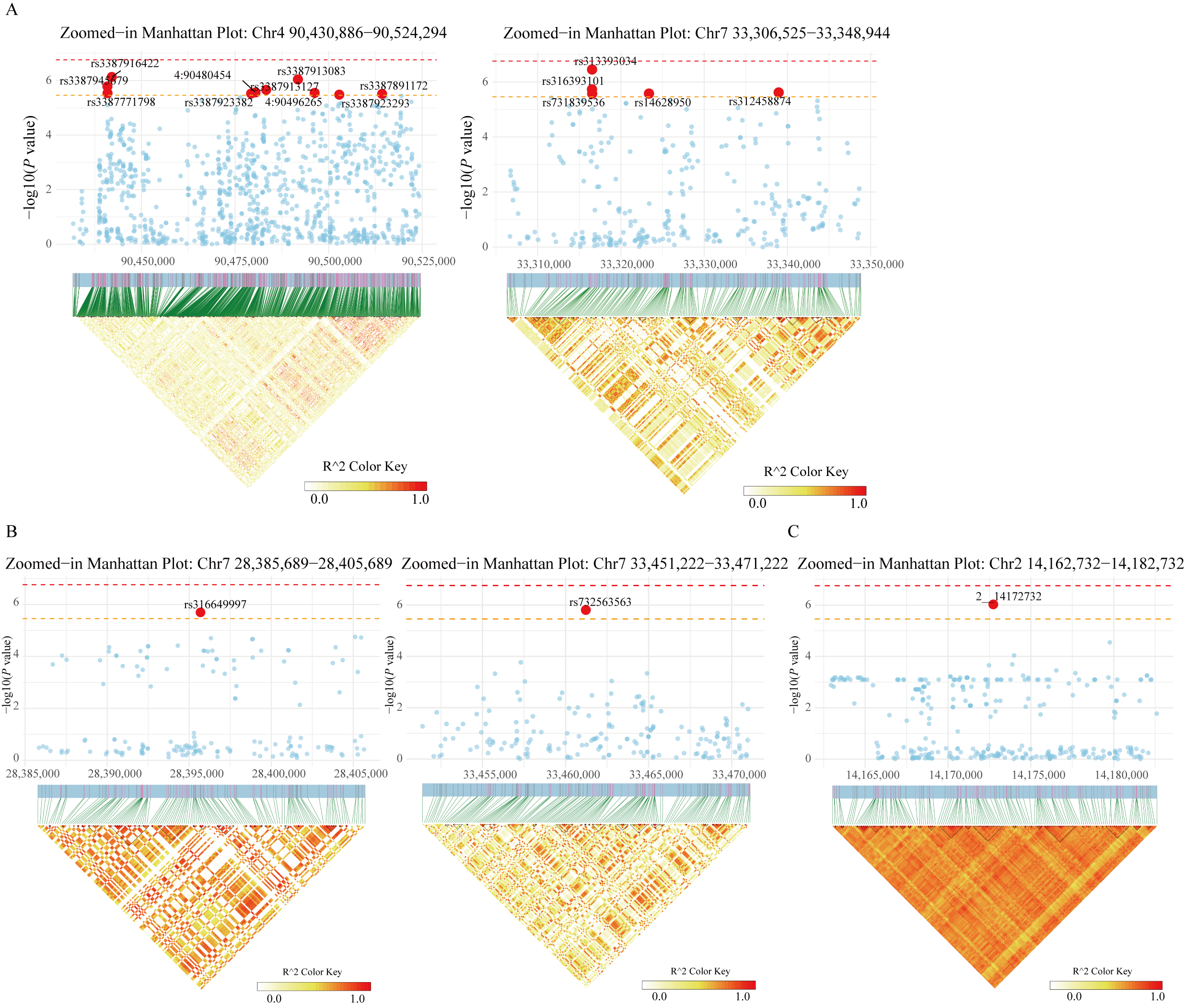

Supplement: Supplementary file 1 [file ijms-26-07876-s001.zip › FIgure S1.tif]

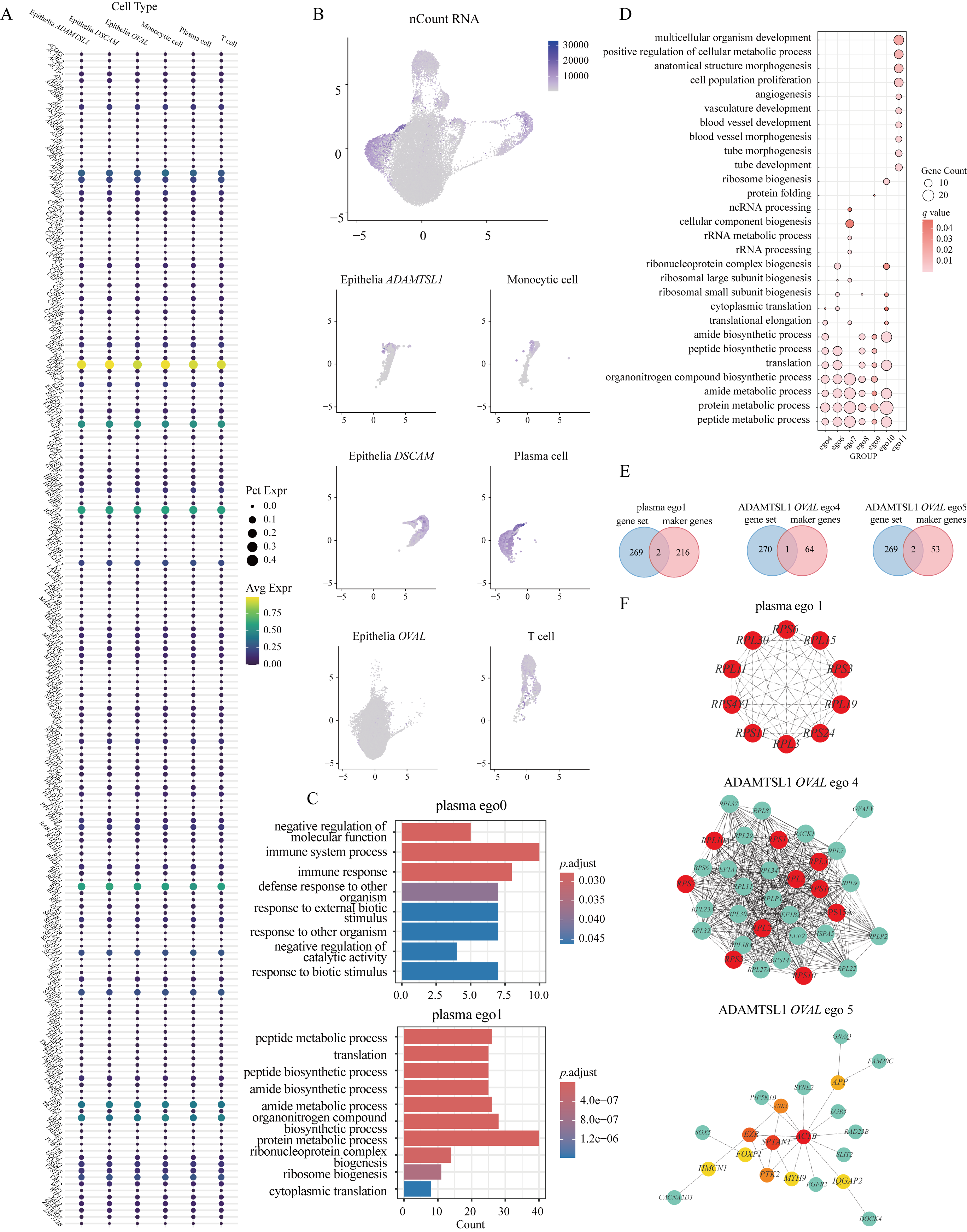

Supplement: Supplementary file 1 [file ijms-26-07876-s001.zip › Figure S2.tif]

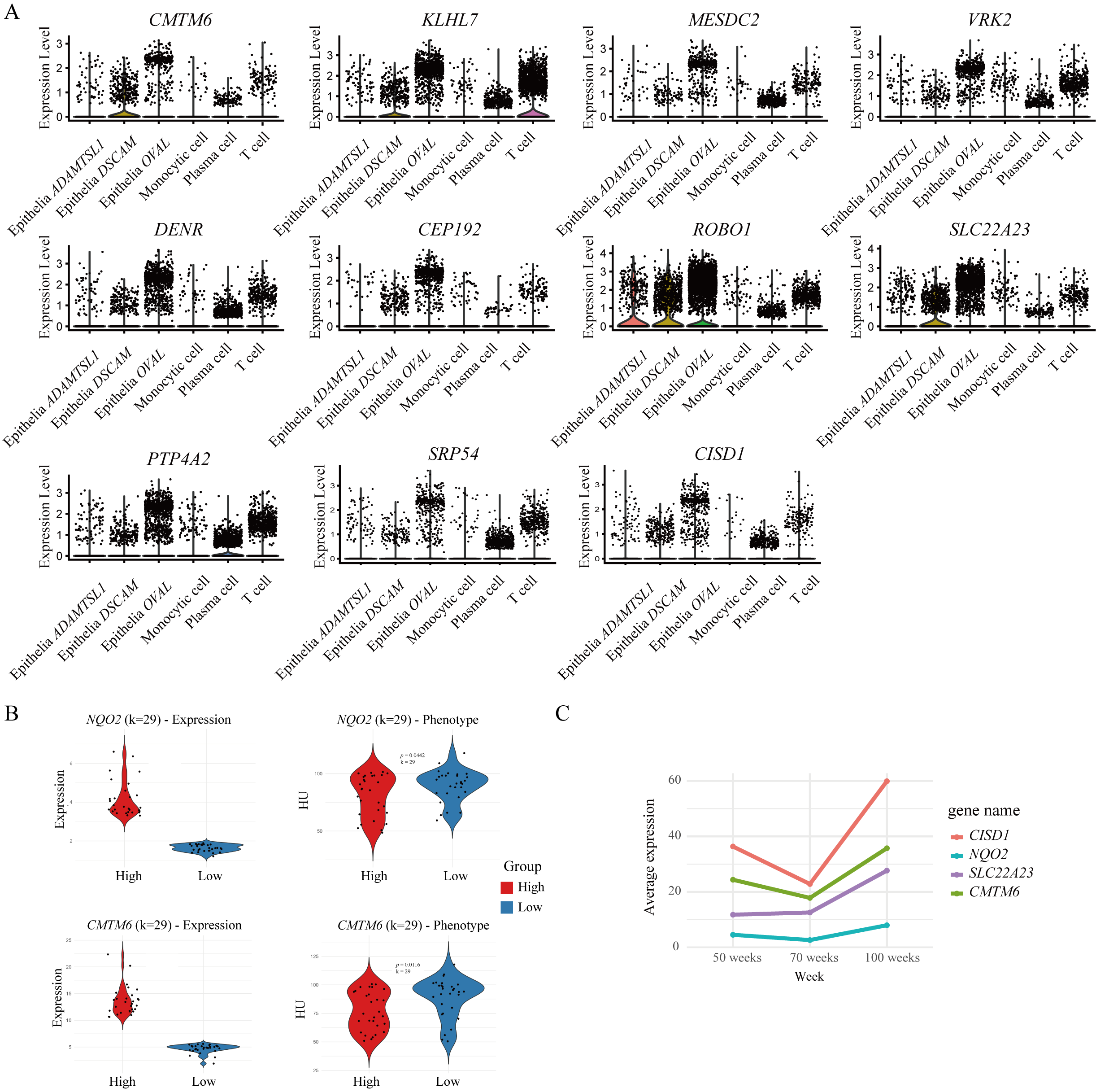

Supplement: Supplementary file 1 [file ijms-26-07876-s001.zip › Figure S3.tif]
